# Supplementary material for: Time- and Spectrally-Resolved Photoluminescence Study of Alloyed CdxZn1−xSeyS1−y/ZnS Quantum Dots and Their Nanocomposites with SPIONs in Living Cells
Source: Int J Mol Sci. 2022 Apr 6;23(7):4061. doi: 10.3390/ijms23074061 (PMC8999546; doi:10.3390/ijms23074061)
Supplement: Supplementary file 1 [file ijms-23-04061-s001.zip › ijms-1643841-supplementary 2.pdf]

International Journal of
Molecular Sciences

MDPI

Supplement

Time- and Spectrally-Resolved Photoluminescence Study of Alloyed $Cd_xZn_{1-x}Se_yS_{1-y}$/ZnS Quantum Dots and Their Nanocomposites with SPIONs in Living Cells

Anna Matiushkina [1,†], Ilia Litvinov [2,*,†], Anastasia Bazhenova [1], Tatiana Belyaeva [2], Aliaksei Dubavik [1], Andrei Veniaminov [1], Vladimir Maslov [1], Elena Kornilova [2,3] and Anna Orlova [1]

Optical properties of QDs

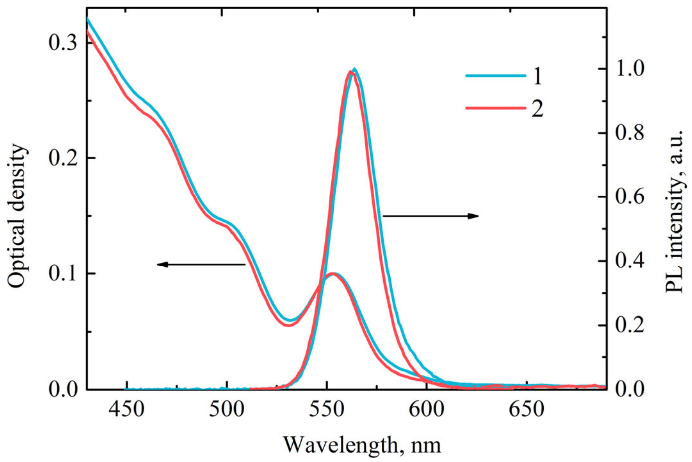

Figure S1. The absorption and PL spectra of solutions of hydrophobic QDs in chloroform (1) and hydrophilic QDs in DMSO (2) after their surface solubilization with L-cysteine. The PL excitation wavelength is 405 nm.

Characterization of QDs by SEM

The QD size estimated by SEM images is about 6 nm. A selected quantum dot is marked with red lines on the SEM image (Fig.S2).

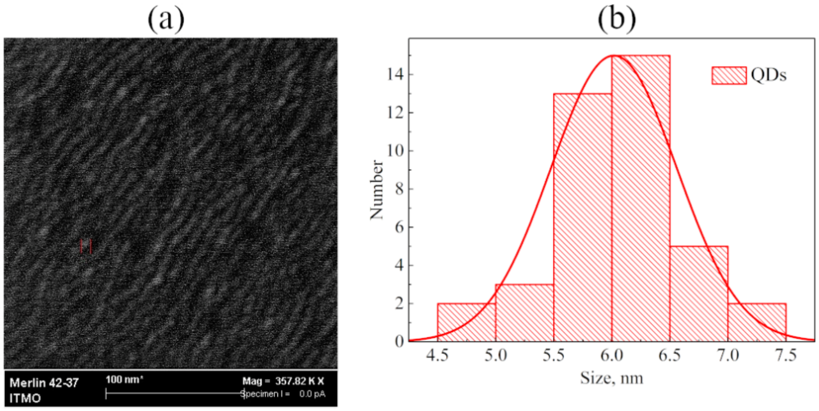

Figure S2. SEM image (a) and size distribution histogram (b) of alloyed ($Cd_xZn_{1-x}Se_yS_{1-y}$)/ZnS quantum dots.

Optical properties of QD-SPIONs

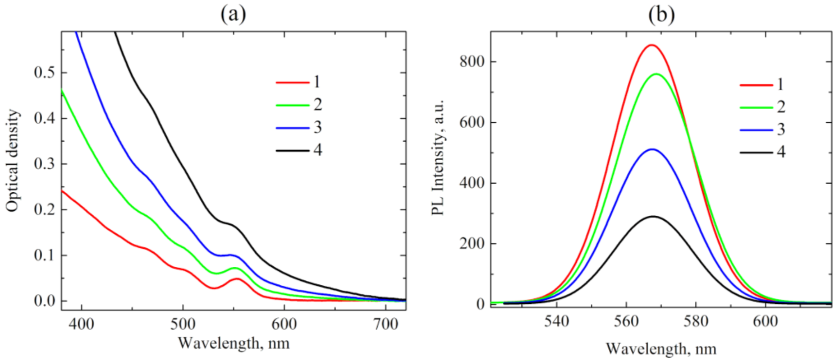

Figure S3. Absorption (a) and PL (b) spectra of samples. The PL excitation wavelength is 515 nm.

PL kinetics of QDs in the QDs-SPIONs

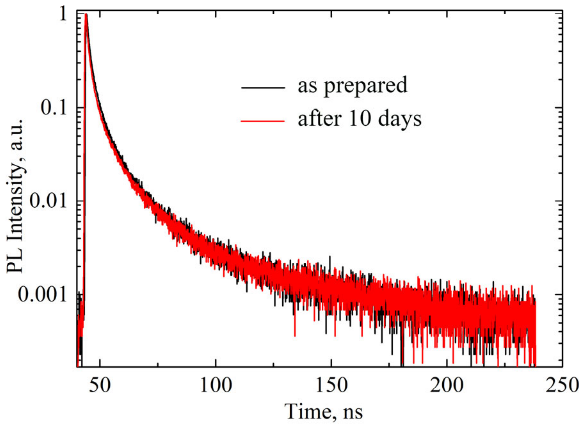

Figure S4. PL decay curves of QDs in the QDs-SPIONs nanocomposites in DMSO over time.

Characterization of nanocomposites by dynamic light scattering

The hydrodynamic size of composites with n=1, estimated by dynamic light scattering during several independent experiments, was found to be about 91 nm (Fig.S5).

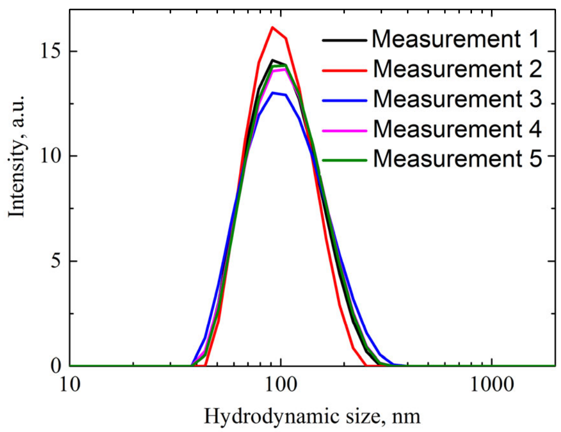

Figure S5. The size distribution of nanocomposites obtained by dynamic light scattering.

Characteristics of SPIONs and their interaction with cells

To produce QDs-SPIONs composites, amphiphilic superparamagnetic Fe_3O_4 nanoparticles stabilized with triethylene glycol were used. The images of SPIONs shown in Fig.S6a were obtained using the TEM. The SPION size distribution based on TEM images (Fig.S6b) shows that the average nanoparticle diameter was approximately 8.5 nm.

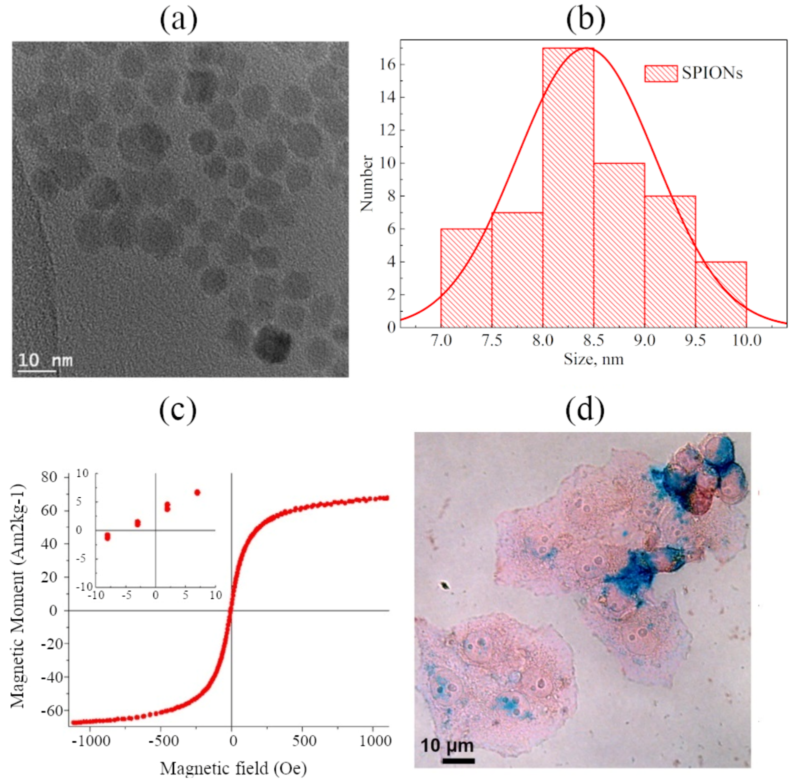

Figure S6. Characterization and visualization of SPIONs: (a) – TEM image; (b) – SPION size distribution histogram obtained from TEM; (c) – magnetization curve; (d) – light microscopy image of HeLa cells with SPIONs (50 nM, 24 h) stained with Perls Prussian blue.

The maximum magnetization value was used to evaluate the magnetic properties of SPIONs. From the sample magnetization curve shown in Fig.S6c, magnetic saturation and almost zero residual magnetization of the sample are observed; therefore, the diagram does not display the hysteresis loop. Thus, we can say that the sample under study behaves as a superparamagnetic one. Similar characteristics of magnetic nanoparticles have been described by other authors. To study the interaction of SPIONs with the cells, they were incubated with SPIONs for 24 h. The presence of iron in HeLa cells was detected using the Perls reaction. The images of the cells with nanoparticles shown in Fig. S6d were obtained by light microscopy.

Blue staining indicates the presence of SPIONs in the vesicular structures, but unlike QDs, slight blue staining was also found in the cytoplasm, thus indicating that small SPIONs can penetrate through the plasma membrane directly (Fig. S6d). In addition, as in the case of QDs, there were clusters of SPIONs located on the surface membrane of the cell. The data obtained are consistent with those available in the literature. It is important that in the areas with the highest concentration of SPIONs, the cells were rounded, which may indicate a toxic effect of SPIONs at high concentrations.
